# Response of Maize (*Zea mays* L.) to Foliar-Applied Nanoparticles of Zinc Oxide and Manganese Oxide Under Drought Stress

**DOI:** 10.3390/plants14050732

**Published:** 2025-02-27

**Authors:** Perumal Kathirvelan, Sonam Vaishnavi, Venkatesan Manivannan, M. Djanaguiraman, S. Thiyageshwari, P. Parasuraman, M. K. Kalarani

**Affiliations:** 1 Department of Agronomy, Tamil Nadu Agricultural University, Coimbatore 641 003, India; sonamvaishnavi123@gmail.com (S.V.); manivannanv@tnau.ac.in (V.M.); parasuraman.p@tnau.ac.in (P.P.); 2 Department of Crop Physiology, Tamil Nadu Agricultural University, Coimbatore 641 003, India; jani@tnau.ac.in; 3 Department of Soil Science and Agricultural Chemistry, Tamil Nadu Agricultural University, Coimbatore 641 003, India; thiyageshwari@gmail.com; 4 Directorate of Crop Management, Tamil Nadu Agricultural University, Coimbatore 641 003, India

**Keywords:** stay green, drought, nanoparticles, maize

## Abstract

Maize (*Zea mays* L.) is an important crop grown for food, feed, and energy. In general, maize yield is decreased due to drought stress during the reproductive stages, and, hence, it is critical to improve the grain yield under drought. A field experiment was conducted with a split-plot design. The main factor was the irrigation regime viz. well-irrigated conditions and withholding irrigation from tasseling to grain filling for 21 days. The subplots include six treatments, namely, (i) the control (water spray), (ii) zinc oxide @ 100 ppm, (iii) manganese oxide @ 20 ppm, (iv) nZnO @ 100 ppm + nMnO @ 20 ppm, (v) Tamil Nadu Agricultural University (TNAU) Nano Revive @ 1.0%, and (vi) zinc sulfate 0.25% + manganese sulfate 0.25%. During drought stress, the anthesis–silking interval (ASI), chlorophyll *a* and *b* content, proline, starch, and carbohydrate fractions were recorded. At harvest, the grain-filling rate and duration, per cent green leaf area, and yield traits were recorded. Drought stress increased the proline (38.1%) and anthesis–silking interval (0.45 d) over the irrigated condition. However, the foliar application of ZnO (100 ppm) and nMnO (20 ppm) lowered the ASI and increased the green leaf area, leaf chlorophyll index, and proline content over water spray. The seed-filling rate (17%), seed-filling duration (11%), and seed yield (19%) decreased under drought. Nevertheless, the seed-filling rate (90%), seed-filling duration (13%), and seed yield (52%) were increased by the foliar spraying of nZnO (100 ppm) and nMnO (20 ppm) over water spray. These findings suggest that nZnO and nMnO significantly improve the grain yield of maize under drought stress conditions.

## 1. Introduction

Maize (*Zea mays* L.) is one of the most versatile and multifaceted crops and has wide adaptability under diverse climatic conditions across the globe. Maize is popularly known as the “Queen of Cereals” owing to its highest genetic yield potential. Currently, 1147.7 million tonnes of maize are produced across 170 countries in an area of 193.7 million ha with an average productivity of 5.75 t ha^−1^ [1]. In India, maize is the third most important food crop after rice (*Oryza sativa* L.) and wheat (*Triticum aestivum* L.). Maize is cultivated over an area of 9.90 million ha with a production of 31.5 million tonnes [2]. In Tamil Nadu, maize is cultivated in an area of 4.26 lakh ha with a production of 29.89 lakh tonnes, with an average productivity of 7007 kg ha^−1^ [3]. Apart from being a staple food crop, maize is also used for manufacturing corn ethanol, important raw materials for poultry and animal feed, green fodder, corn starch, oil, and corn syrup. Other industrial uses of corn oil include the manufacturing of soap, salve, paint, rust-proofing materials, inks, textiles, nitroglycerine, and insecticides. Maize flour is mostly utilized as a carrier material for drug molecules in pharmaceutical preparations [4].

Therefore, there is a growing demand for maize grain across the country, and in the future, the requirements for it will also be higher due to the progressive growth in the poultry sector. As a consequence, the area of maize cultivation in Tamil Nadu is increasing exponentially in both rainfed and irrigated conditions. Nevertheless, the productivity of maize is influenced by abiotic factors like the rainfall, temperature, and wind velocity. The occurrence of early drought during the early vegetative period results in a poor plant population load per unit area. Similarly, the incidence of moisture stress during the flowering stage leads to severe yield penalties due to poor pollen viability, pollen sterility, and abortion. Depending on the severity or duration of drought and crop stages, maize production losses can vary from 30% to 90%. The most vulnerable and affected stages are flowering and grain filling. Drought imposes a water deficit, leading to scarcity in moisture availability and restricted growth and yield. Water deficit stress restricts stomatal opening, accelerates the photoreduction of oxygen in the chloroplast, and increases photorespiration, eventually leading to oxidative damage due to the accumulation of reactive oxygen species (ROS) in plants [5]. The occurrence of severe drought is mainly caused by a rise in the average global temperature that is resulting in a changing climate, which is unpredictable [6]. By 2050, 1.5–1.7 billion people in South Asia are predicted to be impacted by water scarcity [7]. Zinc is one of the most essential elements in carbohydrate metabolism. In addition, zinc activates many enzymatic reactions [8,9]. Zinc application can reduce the effects of drought stress on plant growth by controlling the activity of membrane-bound NADPH oxidase, stopping photooxidative damage, reducing the generation of ROS, and increasing the activities of superoxide dismutase (SOD), peroxidase (POD), and catalase (CAT) involved in detoxifying ROS [10].

Currently, the use of nanomaterials (NMs) in agriculture has increased due to their novel properties, such as their increased surface area and size [11]. Studies have indicated that nanomaterials with antioxidant properties can alleviate abiotic stresses like drought and high temperature [12]. Our research on maize showed that zinc selenide possessed a strong antioxidant property and alleviated reproductive-stage drought in maize [13]. Similarly, nanoselenium alleviated reproductive-stage drought stress by acting as an osmoticum and antioxidant [14]. Similarly, seed priming with nano-zinc oxide particles improved wheat seed germination by 14% compared to its bulk material, namely, zinc sulfate. Along with this, the 1000-grain weight, number of seeds per cob, cob weight, starch, and total soluble protein contents were increased due to nZnO application [11,12,13]. Data on ecotoxicity have shown that most of the nanomaterials used in agriculture are safe [12,13,14]. In another study, it was evident that the foliar application of nZnO @ 100 mg L^−1^ improved the grain yield of maize challenged with salinity stress [15]. Foliar and soil-applied nZnO could improve the yield of maize cultivars under normal conditions [16].

Crop stress management using manganese (Mn) compounds in ionic form has been recently employed to reduce the negative effects caused by drought, harsh temperatures, and salinity. In response to abiotic stress, an adequate supply of Mn has been shown to remediate plant manganese deficiency, induce MnSOD at the transcriptional level to face ROS production, and stimulate manganese-dependent proteins to maintain cell integrity. The foliar application of Mn at 1.46 kg ha^−1^ and Zn at 0.84 kg ha^−1^ as sulfate salts significantly increased the total Mn and Zn uptake compared to the control. Further, foliar applications of B, Mn, Zn, and Fe had limited effects on the grain yield of maize [17]. Spraying manganese nanoparticles (nMnO) at a concentration of 30 ppm improved the growth, yield, and quality characteristics of the common bean [18]. However, the effects of nMnO under drought stress have not been studied in detail. Also, the effects of combined nZnO and nMnO on yield components in maize have not been studied thoroughly. Hence, this study was conducted to quantify the effects of nZnO and nMnO on drought stress alleviation in maize during the reproductive stages.

## 2. Results

### 2.1. Anthesis–Silking Interval (ASI)

Significant differences in ASI were observed for irrigation regimes, foliar sprays, and their interaction. A longer ASI of 0.45 d was recorded under the drought stress condition than the irrigated control. In foliar sprays, the longest ASI was recorded in the control (4.83 d). The shortest ASI of 3.33 days was recorded under the combined application of nZnO (100 ppm) and nMnO (20 ppm) (Table 1).

### 2.2. Per Cent Total Green Leaf Area

Significant differences in the per cent green leaf area were observed for irrigation regimes, foliar sprays, and their interaction. Irrigated plants had a higher total green leaf area (92.5% of total leaf area); however, under the drought stress condition, the total green leaf area decreased to 65% (Table 1). Among the foliar sprays, TNAU Nano Revive @ 1.0% and nZnO (100 ppm) and nMnO (20 ppm) recorded a higher per cent green leaf of 65% and 62%, respectively.

### 2.3. Leaf Chlorophyll Index (SPAD Units)

Among the irrigation regimes, a significantly higher leaf chlorophyll index was observed under the irrigated condition (39.97 SPAD units), while under the drought stress condition, the leaf chlorophyll index was 34.3 SPAD units. Among the foliar sprays, the combined application of nZnO (100 ppm) and nMnO (20 ppm) nanomaterials increased the chlorophyll index more (39.1 SPAD units) than other treatments (Table 1).

### 2.4. Root–Shoot Ratio

There was no significant effect of the irrigation regime on the root–shoot ratio (Table 1). However, the effect of nanomaterials on the root–shoot ratio was significant. The foliar application of combined nZnO (100 ppm) and nMnO (20 ppm) nanomaterials improved the root–shoot ratio (0.177) over water spray (0.137) (Table 1).

### 2.5. Relative Water Content (%)

There were significant effects from irrigation regimes, foliar sprays, and their interactions on the relative water content (%) (Table 1). In the periods between the irrigation regime, drought stress decreased the tissue water content by 25%. Among the foliar sprays, the combined application of nZnO (100 ppm) and nMnO (20 ppm) nanomaterials improved the tissue water content by 19% over water spray (Table 1).

### 2.6. Chlorophyll “a” Content (mg g^−1^ FW)

Significant differences in chlorophyll “a” content were observed for irrigation regimes, foliar sprays, and their interaction. Drought stress decreased the chlorophyll *a* content (1.68 mg g^−1^) compared to the irrigated control (2.17 mg g^−1^). Among the foliar sprays, the combined application of nZnO (100 ppm) and nMnO (20 ppm) nanomaterials improved the chlorophyll *a* content. Under drought stress, the foliar application of nZnO (100 ppm) and nMnO (20 ppm) led to significantly higher levels of chlorophyll *a* content (Table 2).

### 2.7. Chlorophyll “b” Content (mg g^−1^ FW)

A significant variation in chlorophyll *b* content was observed for irrigation regimes, foliar sprays, and their interactions. Drought stress decreased the chlorophyll *b* content (0.52 mg g^−1^) compared to the irrigated control (0.72 mg g^−1^). Among the foliar sprays, the combined application of nZnO (100 ppm) and nMnO (20 ppm) nanomaterials improved the chlorophyll *b* content. Under drought stress, the foliar application of nZnO (100 ppm) and nMnO (20 ppm) led to significantly higher levels of chlorophyll *b* content (Table 2).

### 2.8. Proline (mg g^−1^ FW)

A significant variation in proline content was observed for irrigation regime, foliar sprays, and their interactions. Between the irrigation regimes, a significantly higher proline content was observed under drought stress conditions (1.97 mg g^−1^) over the irrigated control (1.22 mg g^−1^). Among the foliar sprays, the highest proline content was recorded with the combined application of nZnO (100 ppm) and nMnO (20 ppm) nanomaterials (2.13 mg g^−1^) compared to other treatments (Table 2).

### 2.9. Total Carbohydrate (mg g^−1^ FW)

Significant differences in total carbohydrate content were noticed between irrigation regimes. The highest total carbohydrate content (214.2 mg g^−1^) was recorded under irrigated conditions (254.4 mg g^−1^); however, under drought stress conditions, the total carbohydrate content was only 189.4 mg g^−1^. Among the foliar sprays, the highest total carbohydrates were recorded in the plants sprayed with the combined application of nZnO (100 ppm) and nMnO (20 ppm) (Table 2).

### 2.10. Starch (mg g^−1^ FW)

Like the total carbohydrate content, significant differences were noticed for the starch content (Table 2). Between irrigation regimes, drought stress decreased the starch content more than in the irrigated condition. Similarly to the total carbohydrates, the starch content was higher in plants sprayed with the combined application of nZnO (100 ppm) and nMnO (20 ppm) (Table 2).

### 2.11. Seed-Filling Rate (mg^−1^ d^−1^) and Grain-Filling Duration (d)

The results for the seed-filling rate recorded at 65–70, 70–75, and 75–85 DAS and the grain-filling duration at harvest showed significant differences according to irrigation regimes, foliar sprays, and their interactions (Table 3). Between the periods of the irrigation regime, drought stress decreased the seed-filling rate at 65–70, 70–75, and 75–85 DAS (20, 28, and 47 mg^−1^ d^−1^ vs. 9, 21, and 39 mg^−1^ d^−1^, respectively). Among the foliar sprays, the combined application of nZnO (100 ppm) and nMnO (20 ppm) improved the seed-filling rate (21, 33, and 47 mg^−1^ d^−1^ at 65–70, 70–75, and 75–85 DAS, respectively). Similarly, the grain-filling duration was decreased by drought stress, and the foliar application of combined nZnO and nMnO increased the grain-filling duration compared to other foliar sprays (Table 3).

### 2.12. Grain Yield (t ha^−1^)

Significant differences in grain yield were observed for irrigation regimes, foliar sprays, and their interactions. The grain yield under drought stress (6.65 t ha^−1^) was 20% less than that under irrigated conditions. Among the foliar sprays, the combined application of nZnO (100 ppm) and nMnO (20 ppm) improved the grain yield by 19% over water spray. Similarly, under drought stress conditions, the combined application of nZnO (100 ppm) and nMnO (20 ppm) improved the grain yield over other treatments (Table 4).

### 2.13. Stover Yield (t ha^−1^)

The stover yield significantly varied between the main plot treatments, and the highest stover yield was recorded under irrigated conditions (11.60 t ha^−1^) over drought stress conditions (9.85 t ha^−1^). Among the foliar sprays, a higher stover yield was registered for the combined application of nZnO (100 ppm) and nMnO (20 ppm) nanomaterials (12.20 t ha ^−1^); however, the lowest stover yield (8.02 t ha^−1^) was observed with the control (Table 4).

### 2.14. Crop Water Use (kg ha^−1^ mm^−1^)

A total of 475 mm of irrigation water was administered under well-irrigated conditions, whereas for drought stress conditions, three irrigations were withheld, and, hence, 400 mm of irrigation water was provided. Higher crop water use was observed under normal irrigation (17.28 kg ha^−1^ mm^−1^), which was lower than under drought stress conditions (16.62 kg ha^−1^ mm^−1^). Among the foliar sprays, the highest crop water use (18.79 kg ha^−1^ mm^−1^) was recorded under the combined application of nZnO (100 ppm) and nMnO (20 ppm). The lowest water use of 12.85 kg ha^−1^ mm^−1^ was observed in the control (Table 4).

### 2.15. Cost of Cultivation (Rs ha^−1^)

The cost of cultivation did not vary for irrigation regimes, foliar sprays, and their interactions. Numerically, higher values were noticed under irrigated conditions (Rs. 79,050 ha^−1^) than under drought stress conditions (Rs. 78,060 ha^−1^) (Table 5).

### 2.16. Gross Return (Rs ha^−1^)

Differences in gross returns were observed between the main plot treatments, and the highest gross returns were recorded in well-irrigated conditions (Rs. 197,040 ha^−1^) over drought stress conditions (Rs. 159,520 ha^−1^; Table 5). Among foliar sprays, higher gross returns were recorded in combined nZnO (100 ppm) and nMnO (20 ppm) nanomaterials (Rs. 196,800 ha^−1^) (Table 5).

### 2.17. Net Return (Rs ha^−1^)

Net returns were altered by irrigation regimes, foliar sprays, and their interactions. A higher net return of Rs. 117,994 ha^−1^ was registered under well-irrigated conditions than under drought stress conditions (Rs. 81,462 ha^−1^). Among the foliar sprays, the highest net returns of Rs. 117,448 ha^−1^ were registered with the combined application of nZnO (100 ppm) and nMnO (20 ppm) nanomaterials. The lowest net return was observed in the control (Rs. 60,188 ha^−1^) (Table 5).

### 2.18. Benefit–Cost Ratio

The benefit–cost ratio did not vary significantly among irrigation regimes (Table 5). Among the foliar sprays, the foliar application of combined nZnO (100 ppm) and nMnO (20 ppm) nanomaterials increased the benefit–cost ratio to 2.48. The lowest benefit–cost ratio was noticed in the control.

## 3. Discussion

A significant difference was observed between irrigation regimes for the ASI. Drought stress increased the ASI compared to irrigated conditions. The absence of water prior to tasseling will delay the emergence of silk from husks [9,10], resulting in an increased ASI. In contrast, the foliar spraying of Mno and nZnO decreased the ASI difference because the foliar spraying of nanomaterials can improve osmotic adjustment [19]. The increased osmotic potential caused by foliar spraying with nZnO and nMnO might have increased the tissue water content, resulting in the synchronized emergence of the tassel and ear.

Maintaining green leaf areas under drought stress conditions is essential in maintaining the photosynthetic rate. In the present investigation, higher leaf greenness was observed under irrigated conditions, whereas drought stress decreased the green leaf area. The decrease in leaf green area or increased leaf senescence could be associated with increased oxidative damage under drought stress [20]. Studies have indicated that nZnO and nMnO possesses antioxidative properties [21,22]. This was validated in the present study, which showed that nZnO and nMnO possess antioxidant properties because the plants sprayed with these nanomaterials had higher green leaf areas.

The leaf chlorophyll content was decreased under drought stress compared to irrigated conditions because drought stress can reduce chlorophyll biosynthesis by affecting nitrogen uptake [12] and enhancing chlorophyll degradation by reactive oxygen species (ROS) under abiotic stress conditions [23]. Noctor et al. [24] reported that the chloroplast is the primary source of ROS, and under drought stress, enhanced ROS production might occur, which could degrade the chlorophyll pigment. In contrast, the foliar application of nanomaterials like nZnO and nMnO might have decreased chlorophyll degradation by acting as a potent antioxidant [25]. The plants sprayed with nZnO and nMnO had thick leaves, as evidenced by the specific leaf weight indicating that the increased chlorophyll content may be due to a concentration effect rather than degradation [26,27,28].

The root–shoot ratio in maize was not significantly influenced by irrigation treatment. However, nanomaterials significantly influenced the root-to-shoot ratio. The nanomaterials nZnO (100 ppm) and nMnO (20 ppm) improved the root length compared to other treatments, resulting in a higher root–shoot ratio. The increased root length might have favored the uptake of nutrients under drought stress conditions, favoring better root and shoot growth [29].

The maintenance of the osmotic potential of cells is critical for survival under drought stress conditions [30]. In general, the osmotic potential will be increased by the accumulation of osmolyte-like proline under drought stress conditions [31]. An increased proline content was observed under drought stress conditions [32]. The highest proline content was observed in plants sprayed with nZnO (100 ppm) and nMnO (20 ppm) nanomaterials, which could be due to changes in the tissue water content [33,34]. Proline promotes osmotic regulation by balancing cellular structures, removing free radicals, and protecting cellular redox potential. A higher accumulation of proline in plants improves their drought and salinity resistance [35]. Similarly to proline, the starch content indicated that the starch molecules were converted to reducing sugars under drought stress, resulting in increased osmotic adjustment. The foliar application of nZnO and nMnO might have increased the hydrolytic enzymes, resulting in an enhanced accumulation of osmotically active sugar, namely, glucose. A similar observation was made in sorghum under drought stress, with foliar spraying with nanoselenium [19].

An increased seed-filling rate and duration was observed under irrigated conditions over drought conditions. Similarly, nZnO (100 ppm) and nMnO (20 ppm) foliar spraying increased the seed-filling duration and rate owing to delayed leaf senescence, as evidenced by the per cent green leaf area, which could improve the photosynthetic rate. The increase in the seed-filling rate and duration resulted in a larger seed size; thereby, the grain yield was improved. The delayed leaf senescence, coupled with continued photosynthesis, might have resulted in an increased stover yield. The data in the present study indicated that the foliar application of nZnO and nMnO maintained the green leaf area for a longer duration due to its inherent antioxidant property, which has been associated with a higher stover and grain yield under drought stress [36].

Under drought stress, the highest crop water use (19.38 kg ha^−1^ mm^−1^) was recorded in nZnO (100 ppm)- and nMnO (20 ppm)-sprayed plants. This is in accordance with our earlier finding that the foliar application of nanoselenium or nanocerium decreased the transpiration rate and increased the photosynthetic rate in sorghum [37] through the activation of early midday stomatal closure [37]. Thus, the conserved soil moisture and increased photosynthetic rate due to the foliar spraying of nanomaterials might have caused increased water use under drought stress.

## 4. Materials and Methods

### 4.1. Experimental Details

The experimental trial was conducted during the *Summer* 2024 (January 2024 to May 2024) season at Field No. 37, Eastern Block Farm, Department of Agronomy, Tamil Nadu Agricultural University, Coimbatore, to study the response of maize to deficit irrigation and different nanoparticles with regard to drought tolerance, physiological traits, greenness, yield attributes, yield, and economics. The experiment consisted of two main plots and six subplot treatments. The main plot had usual irrigation levels, and the subplot was sprayed with nanomaterials to mitigate drought stress. The nanomaterial used in this study was purchased from Sigma Aldrich, Bangalore, India. The nZnO particle size was <60 nm, and nMnO particle size was 30 nm. The measured average size of the nMnO was 30 nm, and its zeta potential was nearly ~36.5 mV in 10 mmol/L NaNO_3_ at pH 6.5 based on at least three different samples for each measurement. Similarly, the zeta potential of nZnO was −23.5 mV in 10 mmol/L NaNO_3_ at pH 6.5. The main plot comprised two irrigation regimes viz. one that was well irrigated (M_1_) and one withholding irrigation from the tasseling stage to the grain-filling stage [M_2_ (21 days)]. In contrast, the subplot consisted of six foliar spraying applications with nanoparticles, including a control (water spray; S_1_), nZnO nanoparticle @ 100 ppm (S_2_), nMnO nanoparticle @ 20 ppm (S_3_), nZnO (100 ppm) and nMnO (20 ppm) nanocomposite (S_4_), Tamil Nadu Agricultural University (TNAU) Nano Revive @ 1.0% (S_5_), and ZnSO_4_ @ 0.25% and MnSO_4_ @ 0.25% (S_6_). The experiment was carried out in a split-plot design with three replications. Each nanomaterial, as per the concentration required for 1.5 L of water (S_2_:nZnO: 0.15 g; S_3_:nMnO: 0.03 g; S_4_: nZnO: 0.15 g and nMnO: 0.03 g; S_5_: 15 mL, and S_6_: ZnSO_4_: 3.75 g and MnSO_4_: 3.75 g) was added to 100 mL of water and sonicated for 10 min, and 5 mL of Tween 80 was added and again sonicated for 10 min. The solution was made up to 1.5 L to cover 30 m^2^. The nanomaterial was sprayed using a Knapsack hand sprayer with a hollow cone nozzle in the late evening; each plant received ~7.5 mL of spray solution.

### 4.2. Weather Data

**Figure 1:**
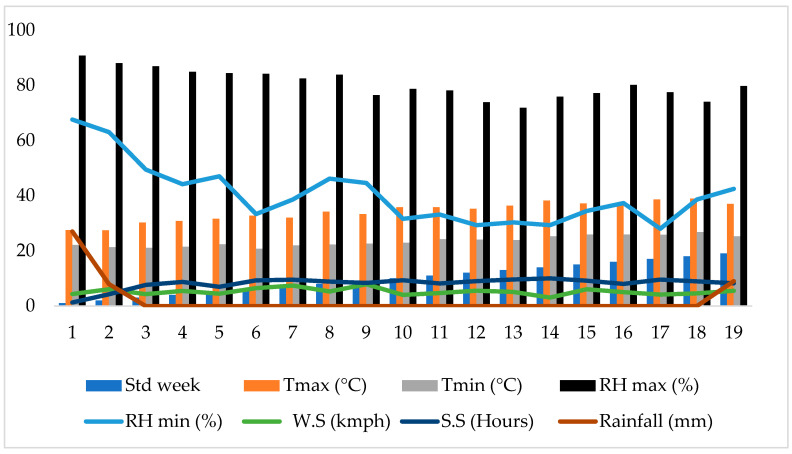
Weather data recorded during winter 2024 and summer 2024 seasons.

The weather parameters recorded during the cropping period revealed that no rainfall was received. The maximum temperature ranged from 29.2 °C to 37.8 °C, while the minimum temperature varied from 21.5 °C to 25.7 °C. The relative humidity observed during the cropping period varied between 73% and 87% and from 32% to 63% during the morning and afternoon, respectively. The average number of daylight hours and the wind speed were 6.5 km h^−1^ and 6.9 h day^−1^, respectively (Figure 1).

The soil of the experimental plots was characterized as a clay loam, with low available nitrogen (162 kg/ha), medium phosphorus (19.7 kg/ha), and high potassium (755 kg/ha). The values for bulk density, particle density, and pore space of the experimental soil were 1.31 (g/cc), 2.23 (g/cc), and 41.08%, respectively [19].

### 4.3. Preparation of Experimental Field

The field was first completely tilled with a tractor-mounted cultivator at the beginning of the experiment, followed by harrowing twice at the optimum soil moisture, and the clods were broken into small soil aggregates to bring the soil to a fine tilth. Following precise land preparation and the ridge-and-furrow method of land configuration, buffer and drainage channels were formed manually. To ensure better germination and uniform crop establishment, high-quality maize hybrid CO H(M)11 seeds were treated with carbendazim at a rate of 2 g kg^−1^ at 24 h prior to sowing, and then they were treated with cyantraniliprole 19.8% + thiamethoxam 19.8% FS at a rate of 4 mL/kg of seed to curtail the incidence of FAW (Fall Army Worm) during the early stages of crop growth. Single seeds were sown hill^−1^ at a desirable soil depth (2–3 cm), and the spacing adopted was 60 cm × 25 cm with respect to inter and intra-row spacing, respectively. Gap filling was conducted 7 days after sowing to maintain the required plant population per plot. All treatments received a common application of farmyard manure (FYM) at a rate of 12.5 t ha^−1^. Additionally, a common fertilizer schedule consisting of 250:75:75 kg N, P_2_O_5_, and K_2_O ha^−1^ was maintained for all treatments. N, P, and K were supplied by urea, single superphosphate, and muriate of potash. Full doses of potassium and phosphorus were applied basally, and nitrogenous fertilizers were applied in three splits viz. 25% of nitrogen basally, 50% at 25 DAS, and the remaining 25% at 45 DAS; a quarter dose of nitrogen was applied basally. On the 25th and 45th DAS, fertilizers were applied as a band placement along the planting rows as the first and second top dressings, respectively. Regarding the irrigation treatments, sowing irrigation was carried out soon after sowing, followed by lifesaving irrigation at 3 DAS, and, subsequently, the third irrigation was carried out at 8 DAS. Thereafter, irrigation was carried out at 6-day intervals. In total, 18 irrigations were performed over the entire cropping period under treatment M_1,_ whereas 14 irrigations were provided for treatment M_2_. No rain was received during the entire cropping period, from sowing to harvesting, and, hence, it was thought to be highly beneficial for the experiment.

### 4.4. Imposing Drought Stress

To impose drought stress in the main plot M_2_ (deficit irrigation), irrigation was stopped from the initiation of the tasseling stage to the grain development stage; in this way, drought stress was enforced continuously for 21 days. Meanwhile, in the main plot M_1_ (the well-irrigated treatment), irrigation was carried out on a regular basis to maintain the soil moisture at field capacity.

During harvest maturity, one border row was harvested first on all four sides of the gross plot area; plants in the net plot were harvested separately; and the collected cobs were dried, dehusked, shelled, and cleaned using a tractor-powered mechanical thresher. The grains were then air-dried further until they reached a moisture level of 14%, and the grain weight in each treatment was assessed separately and expressed in kg ha^−1^. The leftovers (stover) were left in the field for five days to dry out in the sun, and the individual-plot stover yields were noted and expressed in kg ha^−1^. Outs were harvested separately and dried.

### 4.5. Soil Moisture (%) Recorded at 15 cm Depth During the Cropping Period

**Figure 2:**
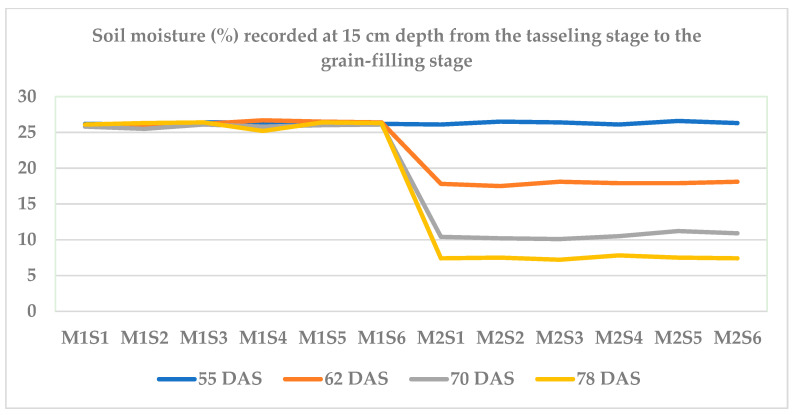
Soil moisture (%) recorded at 15 cm depth during the cropping period (Summer 2024).

Soil moisture was measured with a soil moisture pulse meter (Model PMS-714, Hyderabad, India) at a 15 cm soil depth at 55, 62, 70, and 78 DAS during *Summer* 2024. Under drought stress, the soil moisture depleted gradually from 26.30 to 7.46% from 55 to 78 DAS. However, under irrigated conditions, there was not much variation between treatments from 55 to 78 DAS (25 to 27%). The soil moisture dynamics are presented in Figure 2.

### 4.6. Calculation of Flowering and Anthesis–Silking Interval (ASI)

To calculate 50% silking, the number of days from seeding to the initiation of tasseling for 50% of the population was counted for each plot and expressed in days. Similarly, when at least half of the plant population in the gross plot had visible silk, the date was recorded and considered as the date of 50% silking. Afterwards, the difference in days between female (silk) and male (tassel) flowering was calculated and expressed as the anthesis–silking interval (ASI).

### 4.7. Estimation of Green Leaf Area (% of Total Leaf Area) and Chlorophyll ‘a’ and ‘b’

To calculate the green leaf area, the index leaves (third leaves from the top) of five tagged plants were selected from each net plot area at 65 and 85 DAS, and we measured green leaf area (% of total leaf area) by estimating the percentage of total green leaf area on a scale of 0 to 9, where 0 is 0–10% green and 9 is 90–100% greened. The amount of chlorophyll was measured in the fully expanded third leaf from the top of the main stem. Then, using a spectrophotometer, the amounts of chlorophyll ‘*a*’ and ‘*b*’ content were measured in macerated plant tissue extracted with 80% acetone (by the acetone method) during the tasseling and grain-filling stages. Chlorophyll ‘*a*’ and ‘*b*’ contents were determined using the following formula suggested by Yoshida and Oritani [38], and the results were expressed as mg g^−1^ of the fresh weight of the leaf.

$$\mathrm{Chorophyll}\;a\;=\;\frac{12.7(\mathrm{OD}\;\mathrm{value}\;\mathrm{at}\;663\;\mathrm{nm})\;-\;2.69(\mathrm{OD}\;\mathrm{value}\;\mathrm{at}\;645\;\mathrm{nm})}{1000\;\times \;W}\;\times \;V$$

$$\mathrm{Chorophyll}\;b\;=\;\frac{22.9(\mathrm{OD}\;\mathrm{value}\;\mathrm{at}\;645\;\mathrm{nm})\;-\;4.68(\mathrm{OD}\;\mathrm{value}\;\mathrm{at}\;663\;\mathrm{nm})}{1000\;\times \;W}\;\times \;V$$

where OD is the absorbance at specific wavelengths, V is the final volume of chlorophyll extract in 80% acetone, and W is the fresh weight of tissue extracted.

### 4.8. Leaf Chlorophyll Index (SPAD Unit), Total Carbohydrate (mg g^−1^ FW), Starch (mg g^−1^ FW), and Proline (mg g^−1^ FW)

Using a portable instrument (Model SPAD 502 by Minolta Co., Tokyo, Japan), the leaf chlorophyll index was measured at the tasseling and grain-filling stages in five tagged plants in each plot. Similarly, the proline content of leaves was determined using the [39] recommended method.

The index leaf (the third leaf from the top) was utilized for the estimation of starch content. Consequently, five plants were selected and tagged in the net plot area, then fresh leaves were collected from each plot at 65 and 85 DAS, and the starch content was estimated by taking the OD value, measured using a formula, and expressed as mg g^−1^.

Starch = X * (10/1) * (1/0.5) * (1/1000), where X = Unknown concentration

Similarly for estimating the total carbohydrates, five tagged plants’ index leaves were taken from each plot at 65 and 85 DAS, and the total carbohydrate content was determined by taking OD value, measured using a formula, and expressed as mg g^−1^. The root–shoot ratio was calculated by dividing the dry weight of root biomass by the combined dry biomass of the stem, leaves, and sheath.

### 4.9. Grain-Filling Rate (mg d^−1^), Crop Water Productivity (kg ha^−1^ mm^−1^), and Grain and Stover Yield (kg ha^−1^)

To calculate the grain-filling rate, starting at 15 days after 50% silking, the cobs of designated plants were sampled at five-day intervals until reaching physiological maturity (the formation of a black layer in the placenta). Entire cobs in the gross plot area were harvested separately, dried in a hot-air oven at 60 °C for seven days, threshed thoroughly, weighed, and expressed as mg grain^−1^. Similarly, the grain-filling duration was calculated from 3 days after the 50 per cent silking stage to physiological maturity.

To calculate the crop water productivity, the amount of water delivered in each irrigation was measured with the help of Parshall flumes in all the treatment plots and expressed in mm. Finally, the crop water productivity was calculated for each treatment in all the experimental plots by using the formula below and expressed as kg ha^−1^ mm^−1^.

Crop water productivity = Yield (kg ha^−1^)/Total amount of water supplied (mm)

After each cob was harvested from selected samples from the net plot, cobs were sun-dried, shelled, and cleaned. The grain yield was recorded for the individual treatments after drying to 12 per cent seed moisture and expressed in kg ha^−1^. After harvesting the cobs, the stover in the net plot area was left in the field to sun-dry for 10 days. The dry weight of stover for each treatment was recorded and expressed in kg ha^−1^.

### 4.10. Economics

Based on the current prevailing market prices for critical inputs, hiring charges, and labor wages, the entire total variable cost associated with the treatment from preparatory land cultivation to harvesting and processing was calculated and expressed in Rs ha^−1^. The total income gained from grain and stover yield was computed and expressed in Rs ha^−1^ according to the current market price of maize grain and stover. Once the cultivation costs were subtracted from the gross returns, the net returns were worked out and expressed in Rs ha ^−1^.

### 4.11. Statistical Analysis

The experimental data obtained from main plot and subplot treatments were statistically analyzed using analysis of variance (ANOVA) techniques for the split-plot design. Operational statistics (OPSTAT) were used to compare treatments [40]. The mean comparison was performed by conducting the Tukey–Kramer test at a 5% probability level. Statistical significance is denoted by lowercase letters, indicating that the means with the same letters have no significant difference at *p* = 0.05.

## 5. Conclusions

The major conclusion from this study was that the foliar application of nZnO @ 100 mg L^−1^ and nMnO 20 mg L^−1^ under drought stress delayed the leaf senescence process; thereby, the seed-filling rate and seed-filling duration were increased. The increased source availability resulted in an increased grain yield under drought stress. Therefore, this technology has the potential to reduce the risk associated with terminal drought stress in maize production systems. Before this technology can be widely used, it must be proven to be effective in the long term in non-target organisms, such as beneficial insects, pollinators, earthworms, and other fauna.

## Figures and Tables

**Table 1:** Effect of drought and nanomaterials on anthesis–silking interval (ASI), green leaf area (% of total leaf area), leaf chlorophyll index (SPAD unit), and root–shoot ratio of maize.

Treatments	Anthesis–Silking Interval (ASI) (d)	Green Leaf Area (% of Total Leaf Area)	Leaf Chlorophyll Index (SPAD Unit)	Root–Shoot Ratio	Relative Water Content (%)
Main plot (irrigation regime)					
M_1_ **—Well irrigated	3.71 ^a^	92.50 ^a^	39.97 ^a^	0.152 ^a^	85.2 ^a^
M_2_—Withholding irrigation (21 d) ^#^	4.16 ^b^	65.22 ^b^	34.31 ^b^	0.158 ^a^	63.5 ^b^
SEd	0.03	0.25	0.20	0.005	1.12
CD (*p* = 0.05)	0.12	1.05	0.83	NS	2.15
Subplot (foliar spray)					
S_1_—Control (water spray)	4.83 ^c^	51.67 ^c^	34.7 ^b^	0.137 ^b^	61.5 ^d^
S_2_—nZnO nanoparticle @ 100 ppm *	3.67 ^a^	60.67 ^b^	37.5 ^a^	0.153 ^a^	68.6 ^c^
S_3_—nMnO nanoparticle @ 20 ppm *	3.50 ^a^	60.67 ^b^	36.2 ^b^	0.142 ^b^	71.2 ^b^
S_4_—nZnO (100 ppm) and nMnO (20 ppm) *	3.33 ^a^	62.00 ^b^	39.1 ^a^	0.177 ^a^	75.5 ^a^
S_5_—TNAU Nano Revive @ 1.0% *	4.17 ^b^	60.50 ^b^	36.8 ^a^	0.160 ^a^	66.1 ^c^
S_6_—ZnSO_4_ @ 0.25% and MnSO_4_ @ 0.25% *	4.16 ^b^	65.67 ^a^	38.7 ^a^	0.160 ^a^	63.5 ^d^
SEd	0.14	1.15	1.40	0.011	0.94
CD (*p* = 0.05)	0.30	2.56	3.09	0.025	1.82
Interaction M × S					
SEd	0.18	1.50	1.82	0.015	1.64
CD (*p* = 0.05)	0.41	3.47	4.11	0.039	3.41

^#^ Withholding irrigation from the tasseling stage to the grain-filling stage. * Foliar spraying was carried out 3 days after the imposition of drought stress. ** M_1_. Irrigation was carried out based on field capacity; on average, it was between 5 and 7 days. The means with different alphabet in a column are significantly different at *p* < 0.05.

**Table 2:** Effect of drought and nanomaterials on chlorophyll *a* (mg g^−1^), chlorophyll *b* (mg g^−1^) and proline content (mg g^−1^ FW), total carbohydrate (mg g^−1^ FW), and starch content (mg g^−1^ FW) of maize.

Treatments	Chlorophyll *a* (mg g^−1^ FW)	Chlorophyll *b* (mg g^−1^ FW)	Proline Content (mg g^−1^ FW)	Total Carbohydrate (mg g^−1^ FW)	Starch Content (mg g^−1^ FW)
Main plot (irrigation regime)					
M_1_ **—Well irrigated	2.17 ^a^	0.720 ^a^	1.22 ^b^	214.2 ^a^	170.4 ^a^
M_2_—Withholding irrigation (21 d) ^#^	1.68 ^b^	0.520 ^b^	1.97 ^a^	189.4 ^b^	122.8 ^b^
SEd	0.027	0.007	0.093	0.27	0.48
CD (*p* = 0.05)	0.117	0.033	0.400	1.15	2.06
Subplot (foliar spray)					
S_1_—Control (water spray)	1.05 ^d^	0.35 ^d^	0.88 ^d^	139.1 ^d^	206.4 ^b^
S_2_—nZnO nanoparticle @ 100 ppm *	2.25 ^b^	0.75 ^b^	1.52 ^b^	164.3 ^d^	192.8 ^b^
S_3_—nMnO nanoparticle @ 20 ppm *	1.99 ^b^	0.55 ^c^	1.46 ^c^	170.6 ^c^	193.8 ^b^
S_4_—nZnO (100 ppm) and nMnO (20 ppm) *	2.75 ^a^	0.92 ^a^	2.13 ^a^	254.4 ^a^	161.7 ^a^
S_5_—TNAU Nano Revive @ 1.0% *	1.95 ^b^	0.65 ^b^	1.86 ^a^	244.8 ^b^	168.7 ^a^
S_6_—ZnSO_4_ @ 0.25% and MnSO_4_ @ 0.25% *	1.72 ^c^	0.52 ^c^	1.75 ^b^	237.5 ^b^	178.9 ^a^
SEd	0.140	0.046	0.120	7.75	5.90
CD (*p* = 0.05)	0.312	0.102	0.268	17.2	13.1
Interaction M × S					
SEd	0.182	0.059	0.173	10.01	7.63
CD (*p* = 0.05)	0.419	0.136	0.519	NS	17.0

^#^ Withholding irrigation from the tasseling stage to the grain-filling stage. * Foliar spraying was carried out 3 days after the imposition of drought stress. ** M_1_. Irrigation was carried out based on field capacity; on average, it was between 5 and 7 days. The means with different alphabet in a column are significantly different at *p* < 0.05.

**Table 3:** Effect of drought and nanomaterials on grain-filling rate (mg d^−1^) and grain-filling duration (d) of maize.

Treatments	Grain-Filling Rate (mg d^−1^)			Grain-Filling Duration (d)	1000-Grain Weight (g)
	65–70 DAS	70–75 DAS	75–85 DAS		
Main plot (irrigation regime)					
M_1_ **—Well irrigated	20.5 ^a^	28.83 ^a^	47.50 ^a^	34.44 ^a^	298.9 ^a^
M_2_—Withholding irrigation (21 d) ^#^	9.07 ^b^	21.22 ^b^	39.33 ^b^	30.61 ^b^	284.2 ^b^
SEd	0.53	0.45	0.47	0.31	1.17
CD (*p* = 0.05)	2.32	1.96	2.05	1.34	5.02
Subplot (foliar spray)					
S_1_—Control (water spray)	4.20 ^e^	6.15 ^d^	25.00 ^c^	30.50 ^d^	279.0 ^b^
S_2_—nZnO nanoparticle @ 100 ppm *	17.50 ^c^	22.50 ^c^	41.50 ^b^	31.50 ^c^	290.8 ^a^
S_3_—nMnO nanoparticle @ 20 ppm *	11.50 ^d^	27.00 ^b^	42.50 ^b^	31.83 ^b^	289.2 ^a^
S_4_—nZnO (100 ppm) and nMnO (20 ppm) *	21.00 ^a^	33.50 ^a^	47.50 ^a^	34.50 ^a^	305.4 ^a^
S_5_—TNAU Nano Revive @ 1.0% *	18.50 ^b^	31.00 ^a^	47.50 ^a^	34.33 ^a^	292.8 ^a^
S_6_—ZnSO_4_ @ 0.25% and MnSO_4_ @ 0.25% *	16.00 ^c^	30.00 ^a^	46.50 ^a^	32.50 ^b^	292.2 ^a^
SEd	1.06	1.91	2.95	0.42	7.24
CD (*p* = 0.05)	2.37	4.26	6.58	0.93	16.10
Interaction M × S					
SEd	1.44	2.50	3.83	0.51	9.39
CD (*p* = 0.05)	3.83	5.85	8.74	1.77	21.4

^#^ Withholding irrigation from the tasseling stage to the grain-filling stage. * Foliar spraying was carried out 3 days after the imposition of drought stress. ** M_1_. Irrigation was carried out based on field capacity; on average, it was between 5 and 7 days. The means with different alphabet in a column are significantly different at *p* < 0.05.

**Table 4:** Effect of drought and nanomaterials on grain, stover yield, harvest index, and crop water use of maize.

Treatments	Grain Yield (t ha^−1^)	Stover Yield (t ha^−1^)	Crop Water Use (kg ha^−1^ mm^−1^)
Main plot (irrigation regime)			
M_1_ **—Well irrigated	8.21 ^a^	11.6 ^a^	17.28 ^a^
M_2_—Withholding irrigation (21 d) ^#^	6.65 ^b^	9.85 ^b^	16.62 ^a^
SEd	0.04	0.06	0.33
CD (*p* = 0.05)	0.18	0.24	1.43
Subplot (foliar spray)			
S_1_—Control (water spray)	5.76 ^c^	8.02 ^c^	12.85 ^b^
S_2_—nZnO nanoparticle @ 100 ppm *	7.55 ^b^	10.6 ^b^	17.27 ^a^
S_3_—nMnO nanoparticle @ 20 ppm *	7.43 ^b^	10.5 ^b^	16.99 ^a^
S_4_—nZnO (100 ppm) and nMnO (20 ppm) *	8.20 ^a^	12.2 ^a^	18.79 ^a^
S_5_—TNAU Nano Revive @ 1.0% *	7.95 ^a^	11.8 ^a^	18.22 ^a^
S_6_—ZnSO_4_ @ 0.25% and MnSO_4_ @ 0.25% *	7.69 ^a^	11.2 ^b^	17.58 ^a^
SEd	0.29	0.41	1.21
CD (*p* = 0.05)	0.63	0.91	2.69
Interaction M × S			
SEd	0.37	0.53	1.59
CD (*p* = 0.05)	0.84	1.21	3.77

^#^ Withholding irrigation from the tasseling stage to the grain-filling stage. * Foliar spraying was carried out 3 days after the imposition of drought stress. ** M_1_. Irrigation was carried out based on field capacity; on average, it was between 5 and 7 days. The means with different alphabet in a column are significantly different at *p* < 0.05.

**Table 5:** Effect of drought and nanomaterials on economics of maize.

Treatments	Cost of Cultivation (Rs./ha)	Gross Returns (Rs./ha)	Net Returns (Rs./ha)	Benefit–Cost Ratio (BCR)
Main plot (irrigation regime)				
M_1_ **—Well irrigated	79,050	197,040	117,994	2.49
M_2_—Withholding irrigation (21 d) ^#^	78,060	159,520	81,462	2.04
Subplot (foliar spray)				
S_1_—Control (water spray)	78,050	138,240	60,188	1.77
S_2_—nZnO nanoparticle @ 100 ppm *	78,550	181,200	102,648	2.31
S_3_—nMnO nanoparticle @ 20 ppm *	78,350	178,320	99,968	2.27
S_4_—nZnO (100 ppm) and nMnO (20 ppm) *	79,350	196,800	117,448	2.48
S_5_—TNAU Nano Revive @ 1.0% *	78,800	190,680	111,878	2.42
S_6_—ZnSO_4_ @ 0.25% and MnSO_4_ @ 0.25% *	78,200	184,440	106,238	2.36

^#^ Withholding irrigation from the tasseling stage to the grain-filling stage. * Foliar spraying was carried out 3 days after the imposition of drought stress. ** M_1_. Irrigation was carried out based on field capacity; on average, it was between 5 and 7 days.

## Data Availability

Data is contained within the article.
